# A RAGE-Targeted Antibody-Drug Conjugate: Surface Plasmon Resonance as a Platform for Accelerating Effective ADC Design and Development

**DOI:** 10.3390/antib8010007

**Published:** 2019-01-07

**Authors:** Gareth D. Healey, Asa Frostell, Tim Fagge, Deyarina Gonzalez, R. Steven Conlan

**Affiliations:** 1 Institute of Life Science, Swansea University Medical School, Swansea University, Swansea, SA2 8PP, UK; d.gonzalez@swansea.ac.uk (D.G.); r.s.conlan@swansea.ac.uk (R.S.C.); 2 GE Healthcare Bio-Sciences, SE-751 84 Uppsala, Sweden; asafrostell@gmail.com; 3 GE Healthcare, Little Chalfont, Buckinghamshire, HP7 9NA, UK; tim.fagge@ge.com

**Keywords:** surface plasmon resonance, antibody-drug conjugates, antibodies, gynecological cancers, binding kinetics

## Abstract

Antibodies, antibody-like molecules, and therapeutics incorporating antibodies as a targeting moiety, such as antibody-drug conjugates, offer significant potential for the development of highly efficacious drugs against a wide range of disorders. Despite some success, truly harnessing the superior targeting properties of these molecules requires a platform from which to effectively identify the best candidates for drug development. To streamline the development of antibody-drug conjugates targeting gynecological cancers within our laboratory, we incorporated surface plasmon resonance analysis (Biacore™ T200) into our development toolkit. Antibodies, selected based on positive ELISA screens as suitable for development as antibody-drug conjugates, were evaluated using surface plasmon resonance to determine a wide range of characteristics including specificity, kinetics/affinity, the effect of linker binding, the impact of the drug to antibody ratio, and the effect of endosomal pH on antibody-antigen binding. Analysis revealed important kinetics data and information regarding the effect of conjugation and endosomal pH on our antibody candidates that correlated with cell toxicity and antibody internalization data. As well as explaining observations from cell-based assays regarding antibody-drug conjugate efficacies, these data also provide important information regarding intelligent antibody selection and antibody-drug conjugate design. This study demonstrates the application of surface plasmon resonance technology as a platform, where detailed information can be obtained, supporting the requirements for rapid and high-throughput screening that will enable enhanced antibody-drug conjugate development.

## 1. Introduction

Antibodies and antibody-like molecules offer the potential to develop highly efficacious drugs against a wide range of disorders from cancers to autoimmune diseases to rheumatic and cardiovascular disease. Although the beginnings of this potential have been glimpsed, truly harnessing the superior targeting properties of these molecules requires a platform from which to effectively identify the best candidates for drug development. 

The idea of an immunotherapeutic treatment strategy for cancer emerged in the 1920s focused around the treatment of Hodgkin’s Lymphoma with lymph node extract. However, it is only more recently that immunotherapies have become an established treatment modality, leading to the development of several novel therapeutics for hematological cancers and solid tumors [1]. Over the past 20 years, antibody-based therapies have seen particular success with nearly 20 antibodies gaining US Food and Drug Administration (FDA) approval for use in oncologic care since 1997 [1]. The more recent emergence of chimeric, humanized, and human monoclonal antibodies, has led to a rapid increase in antibody-based therapeutics, which, with 75 Billion USD global sales in 2013, are now the dominant class of molecules within the global biopharmaceutical market [2].

However, antibodies by themselves can, depending on their mechanism of action, display low therapeutic efficacy, meaning alternative approaches are required to increase the potency of antibody-based therapeutics. To address such limitations, antibody-drug conjugates (ADCs) have emerged as a promising therapeutic approach, which combine the selectivity of a targeted treatment with the cytotoxic potency of chemotherapy agents.

The first ADC gemtuzumab ocogamicin (Mylotarg^®^) gained clinical approval in 2000 [3], paving the way for three further ADCs, brentuximab vedotin (Adectris^®^), ado-trastuzumab emtansine (Kadcyla^®^), and Inotuzumab ozogamicin (Besponsa^®^), which were licensed for the treatment of Hodgkin’s and anaplastic large-cell lymphomas, HER-2 positive breast cancer, and relapsed or refractory B-cell precursor acute lymphoblastic leukemia, respectively [4,5,6]. The need to develop efficacious, novel antibody-based therapies means that over 50 different ADCs are currently in preclinical or clinical development [7,8]. 

In such a competitive marketplace, there is an increasing focus on the potential developability of early-stage molecules to prevent costly late-stage failures. This responsibility falls on analytical techniques, which are used to study structural and functional properties including affinity, kinetics, potency, aggregation, solubility, stability, immunogenicity, and pharmacokinetics, as well as cell-based assays to study toxicity and off-target effects.

One such technology, rapidly adopted to study antibody-antigen interactions following its introduction in 1990, is surface plasmon resonance (SPR) [9]. First applied to the study of antibody-antigen interactions and epitope mapping [10], SPR has several advantages over traditional immunoassays such as enzyme-linked immunosorbent assay (ELISA) or radioimmune assay (RIA). It is a label-free technique that monitors the formation and dissociation of biomolecular complexes in real-time, allowing binding kinetics and affinities to be measured. It is also sensitive, requires small sample volumes, works well with crude samples, and has the automation and throughput capability required to support high throughput screening and characterization studies [11,12,13].

Current uses for SPR technology include early-stage screening of hybridoma/phage libraries to monitor expression and triage antibodies based on binding affinity, profiling binding specificity, and providing a detailed understanding of binding kinetics and affinity to characterize antibody-antigen interactions. During therapeutic antibody development, SPR is part of a suite of analytical methods used to study stability, drug-target binding interactions, and binding to Fc receptors, complement and the neonatal receptor (FcRn) to assess the critical quality attributes that determine the efficacy and clinical safety of the final product. As a core technology in analytical and Quality Control (QC) labs, SPR is also used to monitor batch-to-batch variation, support Chemistry, Manufacturing, and Controls (CMC), and as a potency assay to support clinical batch and final product release. 

Given the prevalent use of SPR in the selection and development of standard antibody biotherapeutics, together with its increasing use in many aspects of ADC development, including target selection, antibody kinetics characterization, epitope mapping, and optimization [14,15], we explored how the technology could be used within our lab for the selection and characterization of next-generation ADCs. The importance of the antibody component of an ADC to therapeutic efficacy means that careful consideration must be given to the selection of antibodies for this purpose. Previous work within our laboratory demonstrated variability in the efficacy of antibodies characterized using standard immunoassay techniques such as ELISA, which led us to investigate ADC characteristics using SPR (unpublished). 

Aiming to streamline the design and development of ADCs, we study multiple aspects of effective ADC design, each assessed by SPR. Biacore™ technology is employed to characterize four ADCs that target the Receptor for Advanced Glycation End Products (RAGE), a multi-ligand signaling system that drives innate immune inflammatory responses via nuclear factor-kappa beta (NF-kB) mediated gene activation and is associated with gynecological disease [16]. We demonstrate that a wide range of antibody characteristics can be evaluated including specificity, kinetics/affinity, the effect of linker binding, the impact of drug to antibody ratio (DAR), and the effect of endosomal pH on antibody-antigen binding. In doing so, this study demonstrates the application of SPR Biacore™ technology as a platform, where detailed information can be obtained, supporting the requirements for rapid and high-throughput screening that will enable enhanced ADC development.

## 2. Materials and Methods

### 2.1. Antibody Production

Monoclonal antibody production was performed by Bio-Rad Antibodies (formerly AbD Serotec, Bio-Rad Laboratories, Oxford, UK). All procedures were performed in accordance with the Animals (Scientific Procedures) Act 1986, and the guidance issued in ‘Responsibility in the case of Animals in Bioscience research: expectations of the major research council and charitable funding bodies.’ Monoclonal antibodies against RAGE were produced using standard protocols for monoclonal antibody production [17,18]. Briefly, BALB/c mice, obtained from Charles River, Oxford, UK, were immunized with keyhole limpet hemocyanin (KLH)-conjugated RAGE, or KLH-conjugated peptides corresponding to amino acids (aa) 198–217 or 327–344 of the RAGE protein. Clones were selected based on a positive ELISA screen using bovine serum albumin (BSA)-conjugated peptides. Post-fusion, individual clones were then selected by limiting dilution and clonal expansion to identify genetically stable, antibody-producing cells for subsequent antibody production. One clone with an affinity for the full-length rRAGE protein (RBGO1), two clones with an affinity for aa198–217 (RBGO2 and RBGO3), and one with an affinity for aa327–344 (RBGO4) were selected for antibody production. Antibodies were purified from the tissue culture medium using protein G affinity purification.

### 2.2. Antibody-Drug Conjugation

Murine antibodies against RAGE were reconstituted in 10 mM Tris/HCl (Sigma, Dorset, UK) and 2 mM EDTA (Sigma) pH 8.0. Antibodies were reduced with 3.5 equivalents TCEP:Ab (10 mM in water, Sigma) for 2 h at 37 °C. Without purification the reduced antibody was split in two equal-volume aliquots and each aliquot alkylated with 6.5 equivalents of drug linker: Ab (10 mM MC-ValCit-PAB-MMAE or MC-MMAF, see Figure S1, in DMA with additional DMA added to achieve 5% *v*/*v* final DMA, ADC Biotechnology, St Asaf, UK) for 2 h at 22 °C. Following alkylation, N-acetyl cysteine (Sigma) was used to quench any unreacted toxin linker. The conjugates were purified using a HiTrap™ G25 column (GE Healthcare, Uppsala, Sweden) equilibrated in 5 mM histidine/HCl, 50 mM trehalose (Sigma), 0.01% *w*/*v* polysorbate 20 (Sigma), pH 6.0. Conjugates were analysed by size exclusion chromatography (SEC) for monomeric content and concentration using a calibration curve of naked antibody. Running conditions: Agilent 1100 High Pressure Liquid Chromatography (HPLC), Tosoh TSKgel® G3000SWXL 7.8 mm × 30 cm, 5 μm column (Tosoh Bioscience, Reading, UK), 0.5 mL/min in, 0.2 M Potassium Phosphate, 0.25 M Potassium Chloride, 10% isopropyl alcohol (IPA), pH 6.95. Drug loading of the conjugates was confirmed using a combination of hydrophobic interaction chromatography (HIC) and reverse phase chromatography. HIC was carried out using a TOSOH Butyl-NPR 4.6 mm × 3.5 cm, 2.5 μm column (Tosoh Bioscience) run at 0.8 mL/min with a 12 min linear gradient between A—1.5 M (NH4)2SO4, 25 mM NaPi, pH 6.95±0.05 and B—75% 25 mM NaPi, pH 6.95 ± 0.05, 25% IPA. Reverse phase analysis was performed on a Polymer Lab’s polymeric reversed phase (PLRP) 2.1 mm × 5 cm, 5 μm column (Tosoh Bioscience) run at 1 mL/min at 80 °C with a 25 min linear gradient between 0.05% trifluoracetic acid (TFA)/H_2_O and 0.04% TFA/CH_3_CN. Samples were first reduced by incubation with 1, 4-Dithiothritol (DTT, Sigma) at pH 8.0 at 37 °C for 15 min.

### 2.3. Surface Plasmon Resonance

SPR reagents used were Series S Sensor Chip CM5, HBS-EP+ buffer (10 mM Hepes, 150 mM NaCl, 3 mM ethylenediaminetetraacetic acid, and 0.05% Surfactant P20, pH 7.4), Amine Coupling Kit, Mouse Antibody Capture Kit, including 10 mM glycine-HCl pH 1.7 regeneration solution (all from GE Healthcare).

SPR analysis was performed using a Biacore™ T200 system (GE Healthcare) and HBS-EP+ buffer was used as sample and running buffer. The analysis temperature and sample compartment were set to 25 °C. Immobilization of α-mouse antibody was performed using the Amine Coupling Kit in accordance with the manufacturer’s instructions. The anti-mouse antibody was immobilized in all flow cells, but flow cells 1 and 3 were used as reference cells for antibodies captured in flow cells 2 and 4, respectively. Antibody capture levels were typically in the range 500–1000 RU for the kinetic experiments. Protein or peptide was injected for 60 s in order of increasing concentration over reference and active flow cells, applying a single cycle kinetics procedure using five concentrations. Following each binding cycle, the surface was regenerated with a 180 s injection of regeneration solution from the capture kit, removing the bound antibody. Blank cycles (antibody + buffer injections + regeneration) were performed between each antibody. Data were double referenced by first subtracting responses from the reference flow cell and then subtracting the blank cycles. Data were fitted to a one-to-one binding model using Biacore™ T200 Evaluation Software 2.0. 

### 2.4. Enzyme Linked Immunosorbent Assay (ELISA)

The 96-well micro plates were coated with peptide-BSA conjugates (10 µg/mL) in 20 mM carbonate-bicarbonate buffer (Sigma) at room temperature (RT) for 2 h. After coating, plates were washed (×3) with washing solution, which comprised phosphate buffered saline (PBS; Sigma) containing 0.1% Tween-20 (Sigma). Plates were then blocked with PBS containing 0.1% Tween-20 and 0.2% (*w*/*v*) Gelatin (Sigma) for 30 min at RT. After blocking, plates were washed (×3) with washing solution and doubling-dilutions of primary antibody (10 to 0.0006 µg/mL) prepared in PBS containing 0.1% Tween-20. Primary antibody dilutions were added to the appropriate wells of the plate in 100 µL volumes and plates incubated at RT for 2 h. At the end of the period, plates were washed (×4) with washing solution and horseradish peroxidase (HRP) conjugated α-mouse IgG1 added at a dilution of 1:2000. Plates were incubated at RT for 30 min before washing (×4) and the addition of HRP substrate in accordance with the manufacturer’s instructions (3,3′,5,5′-Tetramethylbenzidine, TMB, liquid substrate system, BD Biosciences, Oxford, UK). After sufficient color development, the TMB substrate reaction was stopped by the addition of 2 M sulphuric acid (BD Biosciences) and plates read at 405 nm using a FLUOstar Omega (BMG Labtech, Aylesbury, UK) spectrophotometer. Data were fitted to a 4-parameter logistic model using MARS data analysis software v3.01R2 (BMG Labtech).

### 2.5. Protein Analysis

Protein or peptides (100 µg/mL) were immobilized onto activated polyvinylidene difluoride (PVDF) membranes (Bio-Rad, Watford, UK) by spotting the desired volume onto the membrane and allowing air-drying at RT. Non-specific sites were blocked with 5% bovine serum albumin (BSA) in tris-buffered saline containing 0.05% Tween-20 (TBS-T) at RT for 1 h. After blocking, membranes were incubated with primary antibody (1 µg/mL) in BSA/TBS-T at RT for 2 h. Membranes were then washed (3 × 5 min) in TBS-T before incubation with HRP-conjugated α-mouse IgG at RT for 1 h. Membranes were then washed with TBS-T (1 × 15 min, 2 × 5 min), then once with TBS (5 min) before visualization using luminol reagent in accordance with the manufacturer’s instructions (Bio-Rad). Images were acquired using a ChemiDoc™ MP imaging system (Bio-Rad) and analyzed with Image Lab™ (Version 3.0) software (Bio-Rad). 

### 2.6. Cell Culture and α-RAGE Antibody Cell Surface Binding and Internalization

The HEC1A (endometrial cancer) cell line was obtained from the European Collection of Authenticated Cell Cultures (ECACC, Public Health England, UK). Cells were grown to 80% confluence before passage in complete medium, which comprised a 1:1 mixture of Dulbecco’s Modified Eagle Medium and Ham’s F-12 nutrient medium (DMEM/F12, Thermo Fisher, Gloucester, UK) supplemented with 10% heat-inactivated fetal bovine serum (FBS, Thermo Fisher), 100 units/mL penicillin, and 100 µg/mL streptomycin (Thermo Fisher). Cells were maintained in a humidified, 5% CO_2_ in air atmosphere incubator at 37 °C, and the culture medium was changed every 48 h.

HEC1A cells were seeded (1 × 10^5^ cells/mL) in 8-well chamber slides (BD Biosciences, Oxford, UK) in 200 µL of stripped medium (complete medium using charcoal stripped FBS) and cultured for 24 h in a humidified, 5% CO_2_ in air atmosphere incubator at 37 °C. After culture, cells were washed in pre-warmed (37 °C) Dulbecco’s phosphate buffered saline (DPBS) and slides were placed on ice. Cells were treated with control medium or medium containing one of the α-RAGE antibodies at 10 µg/mL, and the 8-well chamber slides were incubated on ice for 30 min. Slides were then transferred to the incubator at 37 °C for 240 min, before washing in DPBS and then fixing in 4% paraformaldehyde at 4 °C for 20 min. Where appropriate, cells were permeabilized following fixation, by incubation in 0.01% Triton X-100 in DPBS at 4 °C for 10 min. Cells were then washed and stained with goat anti-mouse IgG-Alexafluor488 diluted 1:1000 in DPBS before nucleus staining with 4′,6-Diamidine-2′-phenylindole dihydrochloride (DAPI).

Images were acquired on a Zeiss LSM 710 confocal microscope (Carl Zeiss Microscopy, Jena, Germany), and analyzed using the Zen 2012 (blue edition) image analysis software v10 (Carl Zeiss). 

### 2.7. RAGE-ADC in vitro Efficacy Screening

HEC1A endometrial cancer cells were seeded (5 × 10^2^ cells/mL) in 96-well tissue culture plates (TPP) in 100 µL of stripped medium and cultured for 24 h in a humidified, 5% CO_2_ in air atmosphere incubator at 37 °C. After culturing was carried out, cells were treated with control medium or medium containing ADCs (0.01–100 µg/mL) for 96 h. Positive controls were cells treated with 0.01% Triton X-100 in stripped medium for the last 4 h of the experiment. Cell growth was monitored over a 96-h period using the RealTime-Glo™ MT Cell Viability Assay (Promega, Southampton, UK) in accordance with the manufacturer’s instructions. Fluorescence was measured using a FLUOstar Omega microplate reader (BMG Labtech, Aylesbury, UK).

### 2.8. Statistical Analyses

Statistical analyses were performed using IBM SPSS Statistics 22 (IBM Corp. Armonk, NY, USA). Initially, the data were tested for homogeneity. Data were analysed by a Student’s *t*-test and are represented mean (SD). A *p*-value < 0.05 was considered statistically significant.

## 3. Results

### 3.1. SPR Provides an Enhanced Platform for Antibody Clone Selection.

To aid the design of novel ADCs for gynecological cancers, we undertook an immunogenicity and sequence alignment analysis of the RAGE protein using the online software tools UniProt and NHLBI-AbDesigner. [19,20] In addition to developing antibodies using the whole RAGE protein, we explored the possibility of designing antibodies against specific regions of the RAGE protein. Specifically, we were interested in targeting a conserved, highly immunogenic region of the RAGE protein, so that subsequent ADCs would be effective against as many RAGE isoforms as possible and the extracellular region adjacent to the transmembrane domain, to enable RAGE targeting following ectodomain shedding. Immunogenicity analysis revealed several highly immunogenic regions within the RAGE protein. We considered all peptides with an NHLBI-AbDesigner immunogenicity score greater than 4.0 and conducted a basic local alignment search tool (BLAST) analysis and sequence alignment of these peptides to identify highly conserved peptides amongst the immunogenic set. This analysis identified the peptide GGDPRPTFSCSFSPGLPRH, corresponding to aa198–217 of the RAGE protein, that was highly conserved amongst human and murine RAGE isoforms and had an immunogenicity score of 10.03. Next, we considered the extracellular region of the RAGE protein that remained following ectodomain shedding (aa317–344) with the aim of identifying a peptide that could be used for immunization. Immunogenicity analysis identified several peptides within this region of the RAGE protein that met our immunogenicity criteria; however, these peptides were not as highly conserved as aa198–217. Based on a balance between immunogenicity and conservation, we selected the peptide GPTAGSVGGSGLGTLALA, corresponding to aa327–344 of the RAGE protein, which had an immunogenicity score of 7.31 and was conserved in eight human RAGE isoforms. 

Subsequently, we generated a small panel of mouse antibodies targeted against RAGE, see Figure S2, selecting antibodies raised against the full-length rRAGE protein (RBGO1); the C1 domain peptide corresponding to aa198–217 of the RAGE protein (RBGO2 and RBGO3) and the transmembrane proximal region peptide corresponding to aa327–344 of the RAGE protein (RBGO4).

**Figure 1:**
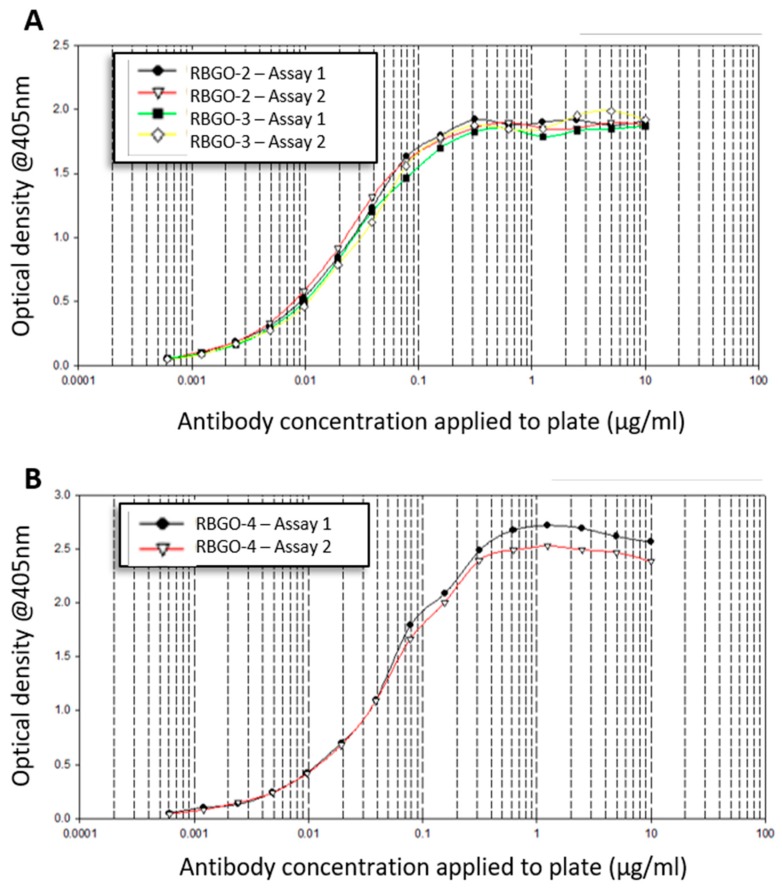
Antibody selection based upon enzyme linked immunosorbent assay (ELISA) against the immunizing peptide. Clones RBGO2 & 3 (**A**) and RBGO4 (**B**) were selected based on a positive ELISA screen using bovine serum albumin (BSA)-conjugated peptides. For ELISA, BSA-conjugated peptides (aa198-217 for RBGO2 and RBGO3; aa327–344 for RBGO4) were immobilized onto micro plates and incubated with two-fold serial dilutions of antibodies. This method represents the standard method of clone selection and good responses to the RBGO2, 3 or 4 antibodies were apparent.

Typically, antibody selection relies on data from ELISA as an indicator of immunogenicity and a criterion for clone selection. Indeed, the antibodies RBGO2, see Figure 1A, RBGO3, see Figure 1A, and RBGO4, see Figure 1B, were selected based on ELISA against the immunization peptides aa198–217 (0.0006 to 10 µg/mL: RBGO2 and RBGO3) or aa327–344 (0.0006 to 10 µg/mL: RBGO4) conjugated to bovine serum albumin (BSA), which showed good immunogenicity and, therefore, grounds for antibody clone selection. 

**Figure 2:**
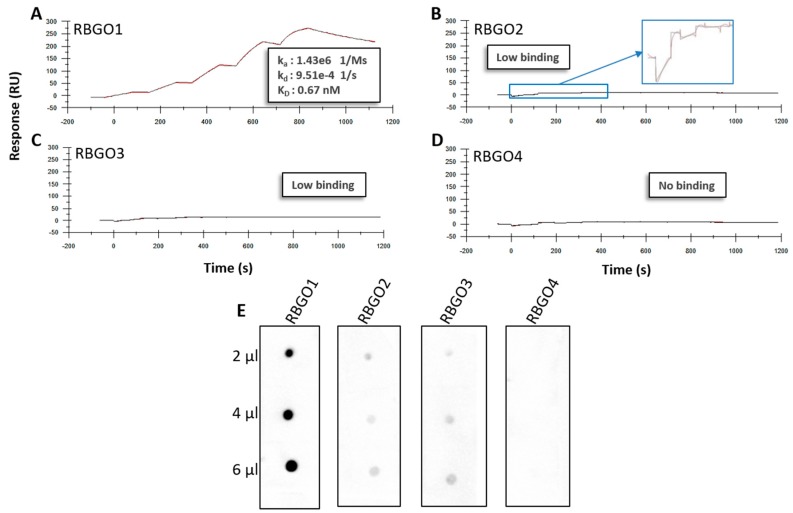
RBGO1 has a higher binding affinity (K_D_) to full-length Receptor for Advanced Glycation End Products (RAGE) protein than RBGO2, RBGO3, and RBGO4. (**A**–**D**) Antibodies were captured to a Sensor Chip CM5 via an amine coupled anti-mouse antibody followed by single-cycle kinetics experiments. RBGO1 (**A**), RBGO2 (**B**), RBGO3 (**C**) or RBGO4 (**D**) antibodies were exposed to recombinant RAGE (2.5 to 200 nM) and data were fitted using a one-to-one Langmuir binding model. Displayed sensorgrams and overlapping fittings are exemplars from three independent experiments and the data shown are the mean. (**E**) Full-length, recombinant RAGE (at the volumes displayed) at 100 µg/mL was immobilized onto activated polyvinylidene difluoride (PVDF) membrane and probed with each of the four α-RAGE antibodies (1 µg/mL). Images were acquired using a Gel-Doc Image acquisition system.

However, we noted that these antibodies had lower lethal dose (LD)_50_ values and reduced internalization rates in HEC1A endometrial cancer cells, compared to an RBGO1-based ADC, when used to make anti-RAGE ADCs, see Table 1. Antibodies raised against peptides can lack affinity to the full-length protein since protein folding can result in the binding epitope being obscured. To explore the reduced efficacy observation, we evaluated antibody binding kinetics to recombinant (r) RAGE protein with a combination of SPR, dot blot analysis, and confocal microscopy. We selected an approach where antibodies were captured onto a CM5 sensor chip via amine coupled anti-mouse antibody, to minimize the time needed for assay development. Kinetic experiments were carried out using rRAGE (2.5 to 200 nM) and the resulting kinetics profiles for RBGO1, see Figure 2A, RBGO2, see Figure 2B, RBGO3, see Figure 2C, and RBGO4, see Figure 2D, showed that binding affinity between the RBGO1 antibody and rRAGE was high, in the picomolar range, whilst binding to the other three antibodies was poor or undetectable. Dot blot analysis using rRAGE, supported the SPR kinetics data, demonstrating the high-binding affinity of RBGO1, weak binding-affinity of RBGO2 and RBGO3, and an absence of binding for the RBGO4 antibody, see Figure 2E. 

**Figure 3:**
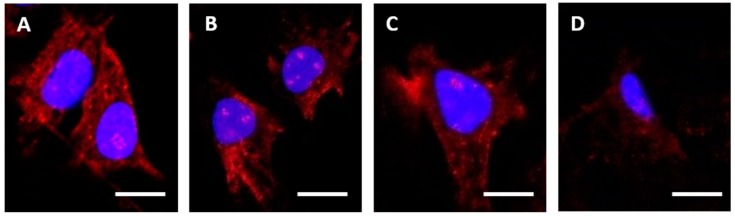
RBGO1 cell surface binding is greater than RBGO2, RBGO3 and RBGO4. (**A**–**D**) HEC1A endometrial cancer cells were incubated in medium containing RBGO1 (**A**), RBGO2 (**B**), RBGO3 (**C**), or RBGO4 (**D**) antibodies at 10 µg/mL for 240 min. After incubation, the cells were washed and fixed. Cell surface bound antibody was imaged via fluorescently labeled secondary antibodies and nuclei stained with 4′,6-Diamidine-2′-phenylindole dihydrochloride (DAPI). Images were acquired on a Zeiss LSM 710 confocal microscope and analyzed using the Zen 2012 image analysis software. Scale bars = 50 µm.

**Figure 4:**
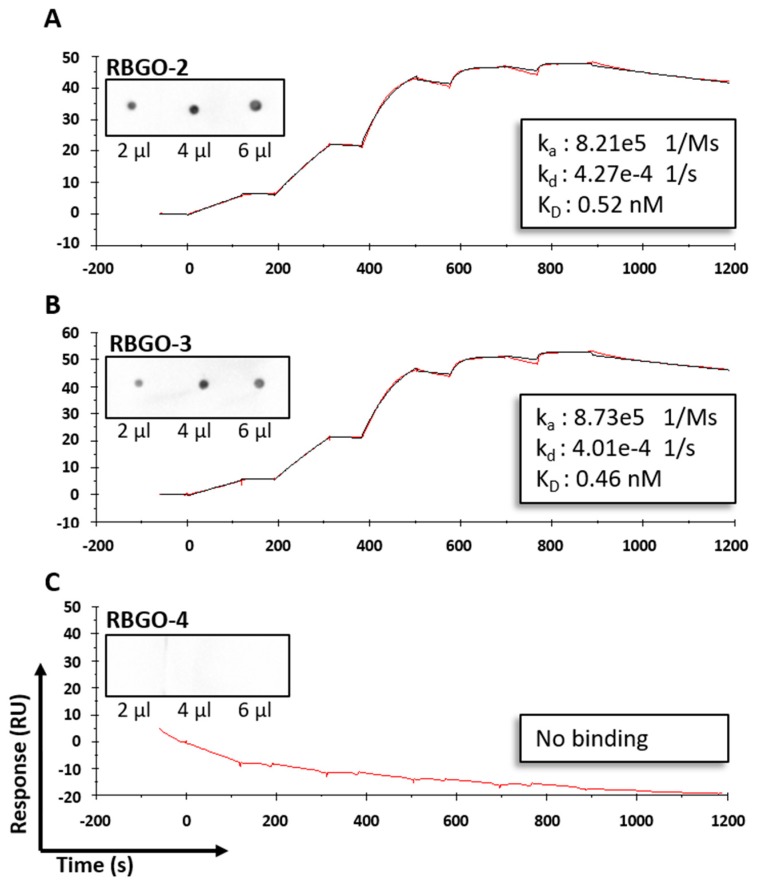
Surface plasmon resonance (SPR) provides an enhanced platform for antibody clone selection. Antibody clones RBGO2 (**A**), RBGO3 (**B**) and RBGO4 (**C**) were assessed for binding kinetics to the immunization peptides used to generate the clones by ELISA. For SPR, antibodies were captured onto a Sensor Chip CM5 via an amine coupled anti-mouse antibody. Single-cycle kinetics experiments were then performed using unconjugated peptides; aa198–217 peptide (RBGO2 and RBGO3; 2.5 to 200 nM) or the aa327–344 peptide (RBGO4; 2.5 to 200 nM). Kinetics were determined using a one-to-one binding model. Curves displayed are exemplar curves from three independent experiments and the data are the mean. Inset images = aa198–217 (**A,B**) or aa327–344 (**C**) were immobilized onto activated PVDF membrane (the volumes displayed at 100 µg/mL) and probed with each appropriate α-RAGE antibody (1 µg/mL). Images were acquired using a ChemiDoc™ MP Imaging system and analyzed with Image Lab™ software.

Additionally, confocal analysis of antibody binding to HEC1A endometrial cancer cells, see Figure 3, corroborated the SPR and dot blot analysis demonstrating strong binding for RBGO1, see Figure 3A, weaker binding for RBGO2, see Figure 3B, and RBGO3, see Figure 3C, and very poor binding for RBGO4, see Figure 3D. Continuing our analysis, we explored the binding kinetics between the RBGO2, 3 or 4 antibodies and their respective peptides, see Figure 4. The RBGO2, see Figure 4A, and RBGO3, see Figure 4B, antibodies bound with high-affinity to the aa198-217 peptide used for clone generation (0.52 ± 0.02 nM and 0.46 ± 0.03 nM, respectively). However, the RBGO4 antibody, did not bind to the aa327–344 peptide used to generate the RBGO4 clone, see Figure 4C. Additionally, we performed a dot blot analysis against the immunization peptides (inset images), which confirmed binding of the RBGO2 and RBGO3 antibodies to the aa198–217 peptide, and the absence of RBGO4 antibody binding to the aa327–344 peptide.

These data highlighted the benefit of validating antibodies raised using specific peptide regions against the full-length target protein in native conditions, a role that SPR is readily amenable to, prior to further development along a therapeutic development pipeline. Additionally, they demonstrate the benefit of adopting a multi-faceted approach to ADC development where multiple technologies are used to give a thorough characterization of ADC candidate antibodies. 

### 3.2. The Effect of Conjugation on Antigen Binding Kinetics is Antibody Dependent.

**Figure 5:**
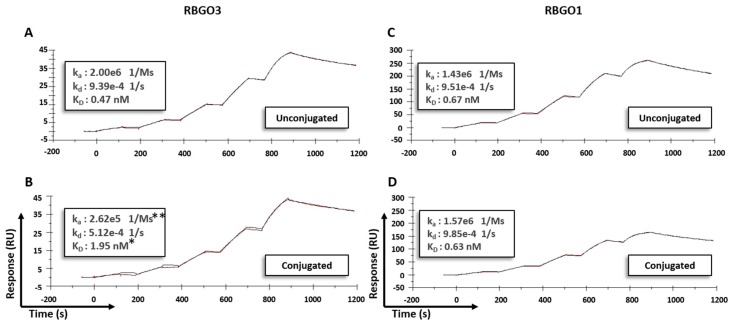
The effect of conjugation on antibody-antigen binding kinetics is antibody-dependent. Antibodies were captured onto a Sensor Chip CM5 via an amine coupled anti-mouse antibody. Single-cycle kinetics experiments were performed. RBGO3 antibody (**A**) and RBGO3-ADC (**B**) were exposed to the aa198–217 peptide (2.5 to 200 nM), and the RBGO1 antibody (**C**) and RBGO1-ADC (**D**) were exposed to rRAGE (2.5 to 200 nM). Kinetics were determined using a one-to-one binding model. Curves displayed are exemplar curves from three independent experiments and data are the mean. * *p* < 0.05, ** *p* < 0.01 compared to the unconjugated antibody.

The production of an ADC requires the conjugation of cytotoxic molecules with a molecular mass in the region of two orders of magnitude smaller than the antibody, via synthetic linkers (again of significantly smaller mass than the antibody), to enable cell killing following cell-surface target recognition and binding by the antibody component of the ADC. Characterization of the effect of antibody conjugation on thermal stability and antigen binding using SPR has previously been described [21]. Whilst different conjugation chemistries vary in their effect on thermal stability, thiol conjugation of IgG1 antibodies is reported to reduce the antibody melting temperature but have no effect on antigen binding in vitro [21]. To investigate the effect of drug-linker conjugation, two of our antibodies, RBGO1 and RBGO3, were conjugated to the antimitotic agents monomethyl auristatin E (MMAE), via a lysosomally cleavable dipeptide valine-citrulline (vc) linker (vcE; see Figure S1A); or monomethyl auristatin F (MMAF), via a non-cleavable maleimido caproyl (mc) linker (mcF; see Figure S1B) and captured onto a CM5 sensor chip via amine coupled anti-mouse antibodies. Characterization of binding kinetics before and after conjugation to vcE was performed using rRAGE (2.5 to 200nM; RBGO1) or aa198-217 (2.5 to 200 nM; RBGO3) and binding/dissociation rates determined using a one-to-one binding model, see Figure 5. Conjugation of the RBGO3 antibody, see Figure 5A,B, resulted in a four-fold reduction in antigen binding affinity (K_D_: conjugated = 1.95 ± 0.03 nM vs unconjugated = 0.47 ± 0.04 nM, *p* < 0.05), whilst conjugation of the RBGO1 antibody had no discernible effect on K_D_ (conjugated = 0.63 ± 0.02 nM vs unconjugated = 0.67 ± 0.03 nM, see Figure 5C,D). Although the dissociation rate was affected by conjugation of the RBGO3 antibody (k_d_: conjugated = 5.12 × 10^−4^ ± 1.3 × 10^−4^ s^−1^ vs. unconjugated = 9.39 × 10^−4^ ± 0.9 × 10^−4^ s^−1^), the predominant factor driving the reduced K_D_ was a ten-fold reduction in the association rate (k_a_: conjugated = 2.62 × 10^5^ ± 1.1 × 10^2^ Ms^−1^ vs unconjugated = 2.0 × 10^6^ ± 2.8 × 10^2^ Ms^−1^ *p* < 0.01). These data suggest that whilst conjugation can impact binding kinetics, as has been previously demonstrated [21], the effect is variable for different antibodies.

### 3.3. The Use of Cleavable or Non-Cleavable Linkers does not Affect Binding Kinetics.

**Figure 6:**
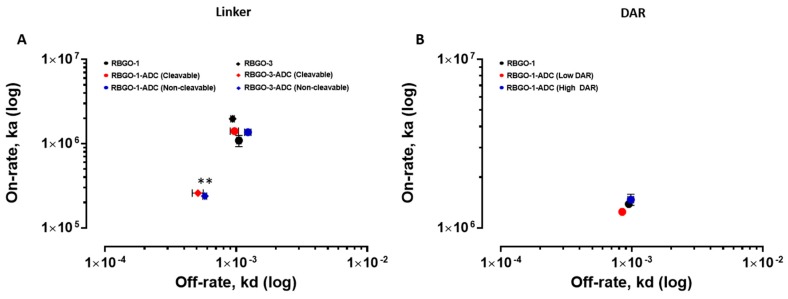
Antibody–antigen kinetics are not affected by the type of linker used or the drug to antibody ratio. Single-cycle kinetics experiments were performed. (**A**) RBGO1 antibody (●), RBGO1-ADC (Cleavable; ● and Non-cleavable; ●) were exposed to rRAGE (2.5 to 200 nM). RBGO3 antibody (◆), RBGO3-ADC (Cleavable; ◆ and Non-cleavable; ◆) were exposed to the aa198-217 peptide (2.5 to 200 nM). (**B**) RBGO1 antibody (●), RBGO1-ADC (Low DAR; ● and High DAR; ●) were exposed to rRAGE (2.5 to 200 nM). On-rates and off-rates, k_a_ and k_d_ were determined using a one-to-one binding model. Data displayed are mean ± SD (*n* = 3). ** *p* < 0.01 for k_a_ compared to the unconjugated antibody.

To further explore the effect of the conjugation described above on binding kinetics, we compared the influence of cleavable and non-cleavable linkers on the antigen binding affinities of the RBGO1 and RBGO3 antibodies, see Figure 6. Both antibodies were conjugated to vcE or mcF and the binding kinetics compared between conjugated and unconjugated forms. Antibodies were captured to a CM5 sensor chip via amine coupled anti-mouse antibodies. Binding kinetics experiments were performed using rRAGE (2.5 to 200 nM; RBGO1) or aa198–217 (2.5 to 200 nM; RBGO3) and binding/dissociation rates determined, see Figure 6A. As previously shown in Figure 5A,B, conjugation of the RBGO3 antibody to vcE or mcF resulted in a reduced k_d_ and significantly reduced k_a_ (*p* < 0.01), whilst no difference in either k_d_ or k_a_ was observed following conjugation of the RBGO1 antibody. These data suggest that whilst conjugation had an impact, the type of linker used did not affect antibody-antigen binding affinity for this set of antibodies. 

### 3.4. Drug to Antibody Ratio does not Affect the Binding Affinity of the RAGE-ADC Lead Candidate. 

The data presented above indicated that the RBGO1 antibody, due to a high binding affinity to native rRAGE, favorable binding kinetics (fast on-rate and slow off-rate) and no loss of these attributes following conjugation, would likely be the most suitable candidate for therapeutic development; therefore, further experiments focused on this antibody alone. RBGO1-ADCs were prepared using varying antibody to TCEP (tris(2-carboxyethyl) phosphine) molar ratios to enable ADCs with low or high drug to antibody ratios (DAR) to be generated. Drug loading of the conjugates was analyzed using a combination of hydrophobic interaction chromatography (HIC) and reverse phase chromatography—Polymer Laboratories Reverse Phase (PLRP). Analysis of the traces (Area Under Curve) indicated average DAR of 1 (low DAR) and 4 (high DAR) were achieved for the test conjugates, see Figure S3. 

Unconjugated RBGO1 antibody, RBGO1-ADC (vcE; low DAR), or RBGO1-ADC (vcE; High DAR) were again captured to the same CM5 sensor chip with amine coupled anti-mouse antibodies that had been used also for the previous analyses. During these experiments we were able to use the same immobilized surface during several weeks, without significant loss of activity, saving time and reagents. Binding kinetics experiments were performed using rRAGE (2.5 to 200 nM) and binding/dissociation rates were determined, see Figure 6B. Using this approach, no significant difference in k_a_ or k_d_ was observed following conjugation at low or high DAR compared to the unconjugated antibody, suggesting that drug loading doesn’t have an impact on antibody-antigen binding for the RBGO1-ADC. In contrast to our observations, previous studies have shown that a high DAR has been shown to affect antigen binding affinity [21,22,23]. However, this effect is variable and importantly, our data demonstrate that a high DAR does not necessarily negatively impact binding affinity and kinetics, together with the applicability of SPR in determining the effect of DAR on antibody-antigen binding. 

### 3.5. The Rate of rRAGE Dissociation from RBGO1-ADC is Influenced by pH, but not by Conjugation.

**Figure 7:**
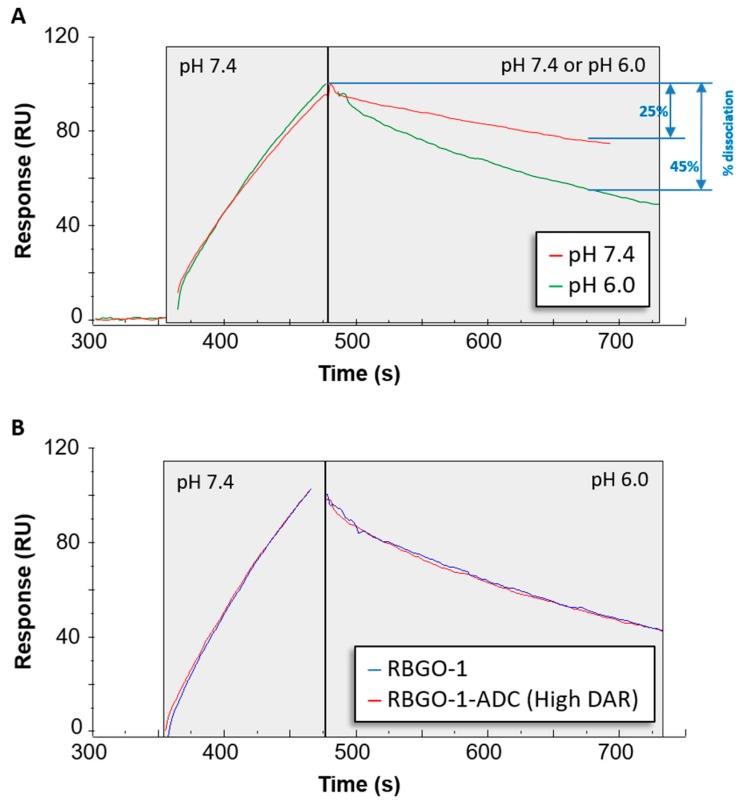
The dissociation rate of the RBGO1 antibody/ADC is increased in acidic pH. Antibody/ADC was captured to a Sensor Chip CM5 via amine coupled anti-mouse antibodies and binding/dissociation experiments were performed using rRAGE (10 nM). Using the dual-inject functionality transition from extracellular to endosomal conditions was mimicked. (**A**) rRAGE was injected in pH 7.4 buffer (extracellular) and allowed to bind to unconjugated RBGO-1, immediately followed by dissociation in either pH 6.0 (endosomal) or pH 7.4 buffer. (**B**) Overlapping sensorgrams showing similar binding profiles for unconjugated and conjugated antibody at the two different pHs. The sensorgrams shown are exemplars from three independent experiments.

The continuous internalization of cell surface receptors into the endosomal compartment of cells is essential to the efficacy of the ADC therapeutic approach, facilitating effective delivery of ADC payload to the internal cell environment where the cytotoxic drug mechanism of action is typically focused. Accordingly, a comprehensive understanding of the molecular mechanisms governing ADC intracellular trafficking is critical to ADC design and selection. Therefore, we wished to investigate the impact of an endosomal-like environment on antigen–antibody dissociation, together with the possible compounding/detrimental effects of conjugation to the binding kinetics of our lead antibody when exposed to endosomal pH. Using the dual-inject functionality in Biacore™ T200, which enables two freely selected solutions to be injected in immediate sequence whilst keeping the same running buffer. Once again, unconjugated RBGO1 antibody or RBGO1-ADC (vcE; High DAR) were captured to the same CM5 sensor chip with amine coupled anti-mouse antibodies that had also been used for the previous analyses. We characterized antibody/ADC association at pH 7.4, whilst determining dissociation kinetics at either pH 7.4 or pH 6.0 with rRAGE (10 nM; Figure 7). The dissociation rate between RBGO1 and rRAGE was increased in a pH 6.0 solution compared to a pH 7.4 solution (% dissociation over 180 s = 45% and 25%, respectively, Figure 7A.), which is in keeping with good ADC design. Additionally, conjugation of the RBGO1 to vcE had no discernible effect on the rate of dissociation at pH 6.0, see Figure 7B. 

## 4. Discussion

Antibody–drug conjugates are a proven example of the type of novel, precision medicines required to combat the increasing incidence of diseases such as the gynecological cancers [24,25]. Aiming to streamline the design and development of ADCs within our laboratory, we evaluated antibodies being developed as ADCs, using SPR technology. This technology enabled us to study ADC characteristics such as specificity, antigen kinetics/affinity, any effects that the pH or our choice of linker type and DAR may have on antigen binding kinetics and demonstrate the applicability of this technology to ADC design and development. SPR analysis within our lab had indicated that antibodies raised against our therapeutic target, RAGE, bound poorly to the full-length protein, data that we verified using dot blot analysis and confocal microscopy. Clone selection based on ELISA against the immunization peptide conjugated to BSA is typical, so we compared ELISA data to kinetics data from SPR analysis using the same peptides. Whilst two of the antibodies in question, RBGO2 and RBGO3, bound with high-affinity to the immunization peptide, the RBGO4 antibody did not, suggesting that the use of ELISA alone might lack the specificity required for the selection of effective, high-affinity binding antibodies for therapeutic development. Indeed, dot blot analysis verified the SPR data, confirming that the RBGO4 antibody did not bind to the aa327-344 peptide. It is unclear why a positive ELISA screen was obtained for the RBGO4 antibody, although interestingly, repeat ELISAs using a non-BSA conjugated aa327-344 or BSA alone were negative, see Figure S4, suggesting that the RBGO4 epitope may span the BSA-peptide junction. 

A variable effect of conjugation on antibody characteristics is known, but this can range from a large effect to none depending on the antibody and conjugation chemistry employed. Our cell-based data demonstrated variability in the cell killing efficacy of our ADCs. An effect that was variable for each antibody and so could not be attributed to the drug or linker being used. Non-specific conjugation often alters the electrostatic properties and hydrophobicity of an antibody with implications for ADC stability and pharmacokinetics [26]. Thiol conjugation, in particular, has a dramatic, DAR-related effect on antibody thermostability compared to alternative techniques such as amine or carbohydrate conjugation; however, the effect is not consistent [21]. Our data showed antibody to antibody variability regarding the effect of conjugation. Although antibody-antigen affinity was comparable for different types of linker and DARs, conjugation caused a four-fold reduction of the affinity (primarily due to a 10-fold reduction in the association rate) for the RBGO3 antibody, whilst the RBGO1 antibody was unaffected despite both antibodies having similar DARs. Utilizing inter-chain, disulfide bridge cysteines is an effective, inexpensive strategy for ADC production. However, variability in the effect of conjugation on antibody binding kinetics means it is important to quantify the impact of conjugation. An important consideration when developing ADCs is also that affinity is not the whole story. ADCs with similar affinity may, in fact, have very different kinetics and, depending on the receptor being targeted, it may be acceptable to have a reduced on-rate if the off-rate is slow enough. The strength of using SPR as a development tool is that affinity can be dissolved into kinetics to obtain the desired kinetic profile for the ADC being developed.

The efficacy of an ADC is dependent on internalization and the release of the cytotoxic payload. Consequently, recent developments in antibody therapeutics have included the design of antibodies that, in addition to binding with high-affinity at the extracellular pH 7.4, also dissociate at a higher rate under endosomal pH 6.0 conditions [27]. Although this concept is yet to be demonstrated for ADCs, such an approach is plausible and could be beneficial for ADCs targeting antibodies that are rapidly recycled back to the cell surface such as HER2 and RAGE [27,28,29]. To explore this aspect of our ADCs, we used an SPR-based method to qualitatively assess the effect of pH on antibody-antigen dissociation. Whilst association was performed at extracellular pH 7.4, it was possible to monitor and compare dissociation at pH 7.4 or endosomal pH 6.0. Interestingly, our lead ADC candidate, based upon the RBGO1 antibody, exhibits high-binding affinity at pH 7.4 and increased dissociation rate at pH 6.0 compared to the dissociation rate at pH 7.4.

## 5. Conclusions

SPR analysis provides an effective platform for the development of ADCs and can be used to assess multiple aspects of these complex advanced biological therapeutic molecules. Of notable value, is the ability to deselect candidate antibodies early in the development process preserving resources that can then be focused on candidates with a greater likelihood of successful development. Using SPR, we were able to determine the specific properties that could explain the superior efficacy of our RBGO1 ADCs compared to others being developed by our lab. The combination of high affinity to the target protein, favorable binding kinetics (fast on-rate, slow off-rate), resistance to loss of binding affinity following conjugation and effective dissociation within an endosomal-like environment, are all key aspects underpinning the efficacy of RAGE targeting RBGO1-ADC and provide a basis for intelligent ADC design. SPR technology has multiple benefits, which when used in combination with alternative approaches, demonstrate its suitability as a key enabler in rational ADC development. 

## Figures and Tables

**Table 1:** Internalization and cell toxicity of Receptor for Advanced Glycation End Products (RAGE) targeting antibody drug conjugates.

Antibody	Internalization (Fluorescence/Cell Area)	ADC	LD_50_ (µM)
RBGO1	0.31 ± 0.04	vcE	0.3 ± 0.02
		mcF	0.09 ± 0.01
RBGO2	0.11 ± 0.02	vcE	2 ± 0.05
		mcF	>100 *
RBGO3	0.12 ± 0.03	vcE	1.5 ± 0.07
		mcF	>100 *
RBGO4	0.03 ± 0.02	vcE	2.4 ± 0.06
		mcF	2.2 ± 0.03

LD_50_ values and internalization, as a function of fluorescence/cell area, of antibody-drug conjugates (ADCs) within HEC1A endometrial cancer cells. * less than 50% cell killing was observed for the range of ADC concentrations tested.

## Supplementary Materials

The following are available online at http://www.mdpi.com/2073-4468/8/1/7/s1, Figure S1: Linkers used during ADC manufacture, title, Figure S2: RAGE antibody binding locations, Figure S3: PLRP traces of high and low DAR ADCs, Figure S4: ELISA of BSA conjugated peptide.
