# Supplementary material for: A RAGE-Targeted Antibody-Drug Conjugate: Surface Plasmon Resonance as a Platform for Accelerating Effective ADC Design and Development
Source: Antibodies (Basel). 2019 Jan 7;8(1):7. doi: 10.3390/antib8010007 (PMC6640708; doi:10.3390/antib8010007)
Supplement: Supplementary file 1 [file antibodies-08-00007-s001.zip › antibodies-404584-suppl-revised/Supplemental figure legends.docx]

**Supplemental figure 1**

Schematic diagram of the relative binding positions of each of the 4 antibodies tested on the RAGE protein.

**Supplemental figure 2**

Conjugation of auristatins to antibodies used cleavable or non-cleavable linkers. MMAE (A) was conjugated via a lysosomally cleavable dipeptide, valine-citrulline linker, which was amenable to protease cleavage. MMAF (B) was conjugated via a non-cleavable, methionine-citrulline linker.

**Supplemental figure 3**

HIC and PRLP analysis confirmed the production of low DAR (A) and high DAR (B) antibody drug conjugates.

**Supplemental figure 4**

The RBGO4 antibody binds to the BSA conjugated aa327-344, but not aa327-344 or BSA alone. The binding affinity of RBGO4 was determined by ELISA. RBGO4 antibody (10 to 0.0006 µg/ml) was immobilised on microplates and exposed to BSA (10 µg/ml), aa327-344 (10 µg/ml) or aa327-344-BSA (10 µg/ml). Data displayed are mean of three independent experiments. Error bars have been omitted for clarity.
