# Supplementary figures and images for: A RAGE-Targeted Antibody-Drug Conjugate: Surface Plasmon Resonance as a Platform for Accelerating Effective ADC Design and Development
Source: Antibodies (Basel). 2019 Jan 7;8(1):7. doi: 10.3390/antib8010007 (PMC6640708; doi:10.3390/antib8010007)

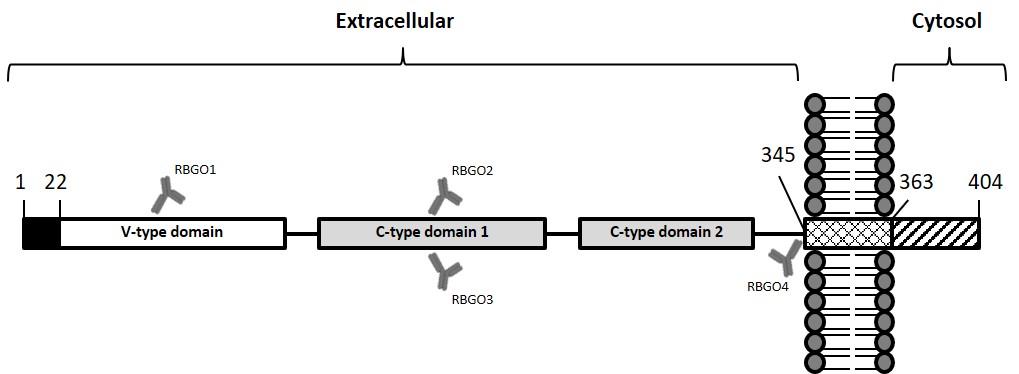

Supplement: Supplementary file 1 [file antibodies-08-00007-s001.zip › antibodies-404584-suppl-revised/Sup Fig 1.jpg]

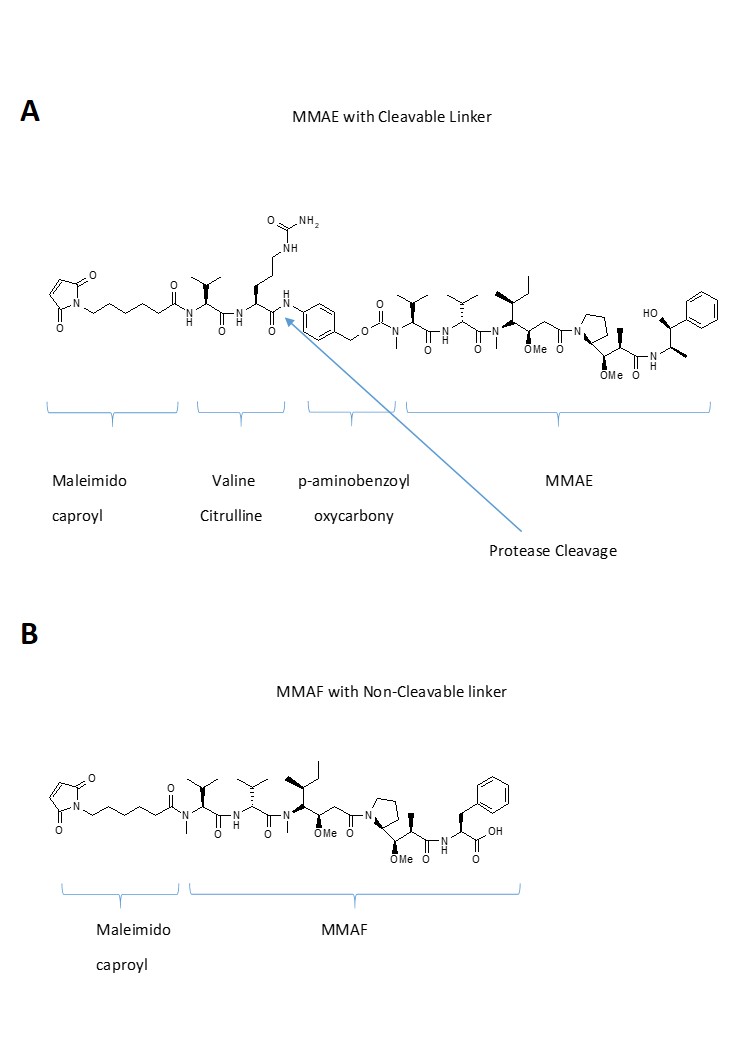

Supplement: Supplementary file 1 [file antibodies-08-00007-s001.zip › antibodies-404584-suppl-revised/Sup Fig 2.jpg]

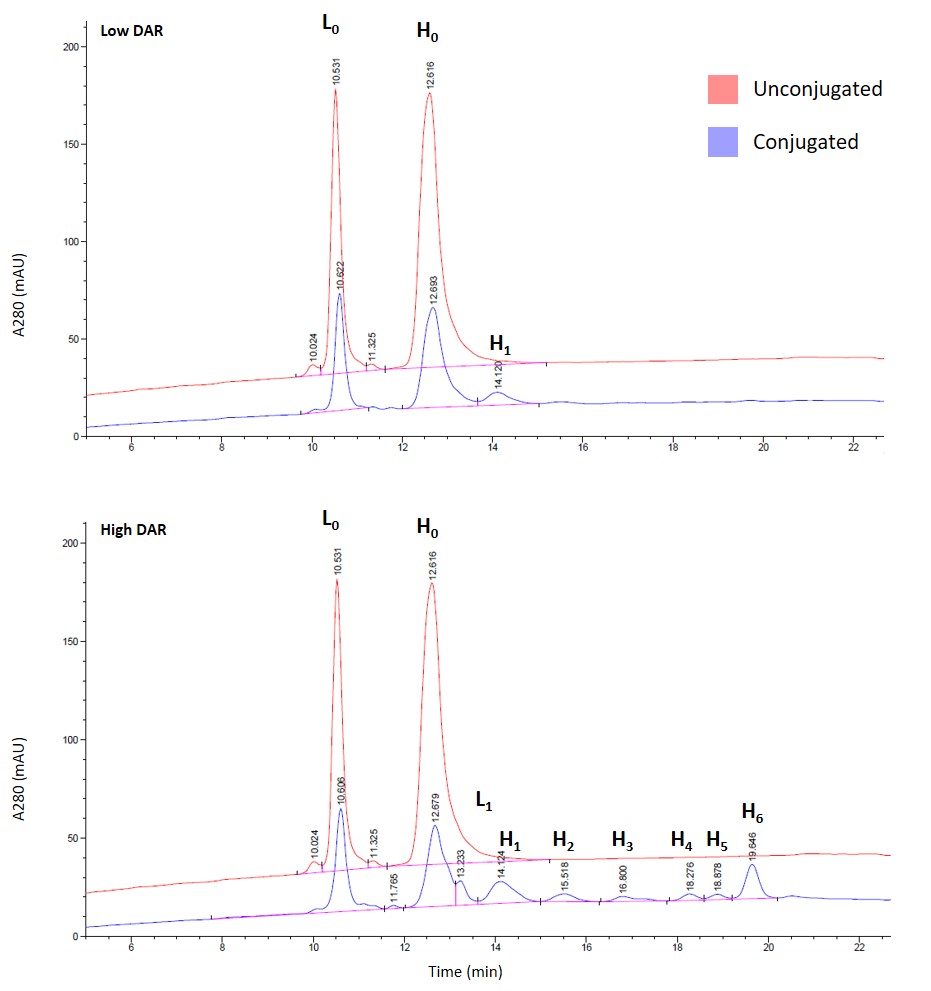

Supplement: Supplementary file 1 [file antibodies-08-00007-s001.zip › antibodies-404584-suppl-revised/Sup Fig 3.jpg]

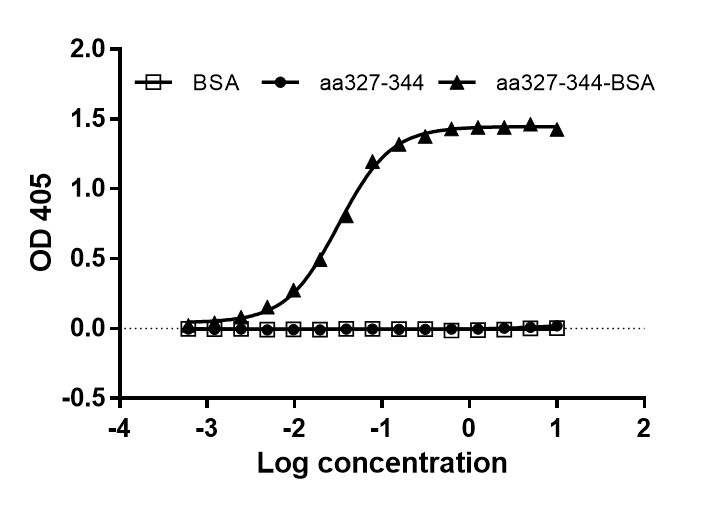

Supplement: Supplementary file 1 [file antibodies-08-00007-s001.zip › antibodies-404584-suppl-revised/Sup Fig 4.jpg]
